# Experiments and Observations upon the Fifth, Seventh, and Eighth Pairs of Nerves

**Published:** 1823-06

**Authors:** S. D. Broughton

**Affiliations:** Member of the Royal College of Surgeons; one of the Surgeons to the St. George's and St. James's Dispensary, and Surgeon to his Majesty's Second Regiment of Life Guards.


**Art. II.**—*Experiments and Observations upon the Fifth, Seventh, and Eighth Pairs of Nerves.* By S. D. Broughton, Esq. Member of the Royal College of Surgeons; one of the Surgeons to the St. George's and St. James's Dispensary, and Surgeon to his Majesty's Second Regiment of Life Guards.

The individual and separate functions of the nervous system form a subject in physiology, which at present excites a more than ordinary degree of interest; and the novelty of some experiments lately published, together with the extreme difficulty attending an accurate discrimination in the results of all inquiries respecting the phenomena of the brain and nerves, has very naturally produced some difference of opinion.

The following simple experiments were made for the purpose of satisfying the author's mind, by actual examination of some points, which had previously undergone a strict scrutiny in much abler hands; and, although they throw no new light upon the subject, they afford some additional evidence in favour of that which has shone from other quarters; in which point of view they may be interesting, if not useful to science.

Upon this, as upon former occasions, the author availed himself of the kindness, zeal, and anatomical skill, of Mr. John Field, in Oxford-street; who is always ready to devote himself to all physiological pursuits, which the nature of his profession affords peculiar opportunities of enjoying.

*Experiment* 1.—The facial branch of the fifth pair of nerves was exposed, just as it emerges from the foramen, and detached by a blunt needle passed under it. The subject of the experiment was an ass, of one year old. By touching and dividing the nerve, signs of great sensibility were manifest. The superior divided end retained its sensibility, and the lower was quite insensible: the division, therefore, was complete. The third branch, on the same side, was similarly divided. The lips on this side lost their sensibility, and their natural tone and adjustment. The ala nasi was also rendered insensible, but the nostril retained its movements in respiration. The branch of the seventh pair, called the communicans faciei, was next exposed, on the posterior edge of the masseter muscle, under the zigomatic process, on the same side. There was not observed any difference between the sensibility of this nerve and the other two. The nostril, previously in full action, immediately ceased to move, and became collapsed; and the lips and muscles on the other side of the face were drawn away, as if deprived of their antagonists, and the animal could eat only on the sound side. A kind of sneezing expression marked the countenance, from the incapability of the ass to regulate its features by a proper adjustment of the muscles on the side submitted to experiment.

*Experiment* 2.—The first experiment being on the left side, the right was tried on the following day. The nerves were divided just as before, with the same results. Both nostrils were now collapsed and motionless, and, with the lips, had lost their sensibility. The ass had no power to pick up its corn, and the under lip hung flaccid and motionless away from the teeth.

*Experiment* 3.—An aged horse was thrown, and the portio dura exposed as close as possible to the edge of the parotid gland, under the ear. In the progress of the dissection, three branches were seen coming out upon the face, the lowest and largest being about the third of an inch in width. They were each successively pinched and pricked, and they all exhibited signs of sensibility. On dividing the two upper branches, nothing occurred; but, when the lower branch was cut in two, the nostril below immediately ceased to move, and became closed, and the sensibility of all the parts below remained entire. The lips on this side seemed to have lost their power of motion, being drawn towards the other. The same operation was performed on the opposite side of the face; and then both nostrils remained closed and motionless, but retained their sensibility. The lip fell from the teeth of the lower jaw loose and flaccid, and sensation continued in both. The animal had no power to pick up its corn. The facial branches of the fifth nerve were successively cut through on each side; when the nostrils and upper lip lost their sensibility, the lower only retaining it.—Mr. Brodie and Mr. Cutler were present at this experiment.

*Experiment* 4.—An aged horse was thrown, and the two lower of the three branches above described were cut through, as close as possible to the parotid gland. The uppermost branch was left entire, being supposed to belong to the fifth pair. The sensibility of the divided nerves was satisfactorily ascertained; but it was now particularly noticed, that at one moment they appeared sensible, and at another not so; and this seemed to depend upon whether their upper or lower edges were touched with a needle. Immediately after division, the nostril collapsed, and sensibility remained. The same division was made on the opposite side, with the same results. The lip fell from the lower jaw, and the animal could only eat by the general movement of the jaw, but could not pick up food.

There appearing to be some uncertainty about the three facial branches, so near each other, in front of the parotid gland, the horse was killed, and these branches traced to their origins; when the circumstances of their sensibility were explained. The two lower branches were found to belong to the trunk of the portio dura, which, imbedded in the gland, bifurcates immediately on emerging from it, each branch of the nerve being accompanied, in the same sheath, by one from the fifth pair.

They are easily separable with the fingers without injury to either, not being interlaced. The uppermost, and smallest of the three, is a branch of the fifth alone. It now became necessary to divide the portio dura where it passes alone through the gland, before its bifurcation and junction with branches of the fifth nerve.

The division of these three ramifications does not affect the sensibility of the nostrils and lips, because these parts are otherwise supplied by the fifth nerve; but the division of the lower two is attended with loss of motion, from the influence of the seventh nerve being cut off from the nostrils and lips altogether. The fasciculus formed by the junction of the seventh and fifth nerves, as they pass from the edge of the gland over the angle of the jaw, sufficiently accounts for the supposed sensibility of the portio dura, and the apparent inconstancy of the evidence of sensation mentioned in the fourth experiment, as well as the minor degree of sensibility ascribed to this nerve by others. Probably, many contradictory testimonies relative to nervous functions may be attributed to similar circumstances.

*Experiment* 5.—An aged horse was thrown, and the parotid gland was divided, in order to expose the trunk of the portio dura unblended with any other nerve. To avoid the great bleeding which attends the wounding of the gland with a knife, the nerve was insulated by the fingers and blunt instruments, and got at with some difficulty. It was transfixed with a pin, pinched, and cut through slowly with scissars, and not the slightest sign of sensation was manifested. The nostril immediately collapsed, and its sensibility remained entire. The lips on this side appeared loose and flaccid, and fell away from the teeth; they retained their sensibility, and the mouth was drawn on one side, its corresponding muscles having lost their motion. The three branches were then divided on the other side, and the paralysis was rendered complete, without any diminution of sensibility.—Mr. Brodie witnessed this experiment.

*Experiment* 6.—The same horse was thrown next day, and the par vagum exposed in the neck on one side, and insulated from its cellular connexions, but carefully retained in its place. It was repeatedly transfixed with a pin, pinched, and slowly cut with scissars, and not the slightest degree of sensation was manifested. When pulled out from its natural position, or squeezed by the forceps, the animal appeared to wheeze, as in obstructed respiration, but exhibited nothing like the twitches and startings which peculiarly mark the production of pain in irritating sensible nerves. This experiment was not repeated, because it confirmed similar observations made in performing numerous experiments on the eighth pair of nerves, for objects of a different kind.

These experiments have been thus fully and minutely detailed, because truth cannot be rendered apparent without a full view of all the circumstances collectively. It will be observed that the different trials were of the same kind, and produced the same results, having been varied only in the order of their succession.

Physiologists may judge for themselves whether the facts detailed bear out the following conclusions:

1. The fifth pair of nerves is highly sensible, and bestows sensibility to the integuments of the face and lips generally; and, when divided, all sensibility ceases in the parts below the division to which this nerve distributes branches; and thus the lips lose their natural degree of tone and adjustment, but are not rendered altogether motionless; and the nasal cartilages continue their action in hurried breathing, although they are deprived of feeling.

2. The portio dura of the seventh nerve is entirely devoid of sensibility, and conveys none to the parts supplied with its branches. It influences the muscles of the face and cartilages of the nostrils, and the actions of the lips; for, when this nerve is divided, all those parts to which its branches are distributed become paralysed. If divided on one side, then the face is drawn to the opposite side, the muscles having lost their balance of action in the proper adjustment of the countenance,—sensibility still remains entire; but the lips, having been deprived of their moving power, hang loose and inactive, the lower lip falling quite away from the teeth, and exposing the gums to view.

3. The par vagum is entirely insensible. If compressed strongly or divided on one side, the wheezing produced indicates oppression in breathing, probably arising from loss of motion in some moving organs of respiration. When the division is made on both sides, this effect is more strongly marked, and becomes fatal.

4. So far as the above experiments go, they tend to prove that nerves have separate properties, and that these are in accordance with the different individual functions belonging to those organs to which their branches are distributed. They partly confirm experiments and observations already before the public, indicating this curious fact, that one set of nerves conveys the impressions of feeling or sensibility, and another influences motion; and that these properties are held individually and independently of each other. The peculiar purposes for which such a distinction may be designed appear to be open, as they have indeed given rise to much ingenious theory and speculation.

The author is aware that the sensibility of the eighth pair of nerves is questioned; and that, in opposition to his conclusion on this point, it is said that the sensibility of nerves varies in degree and at different times. It has also been said that, as the larynx is highly sensible, it is to be presumed that the par vagum, which distributes branches to it, is a sensible nerve.

In answer to these objections, it may be observed that the result of the trials in the sixth experiment left no doubts on the minds of those present whatever: they were most decided in this case, and confirmed many repeated observations formerly made upon the same point, arising from phenomena too constant to admit suppositions and surmises to overbalance them. Whilst other nerves around appeared highly sensible, the par vagum, at the same time, exhibited not the slightest degree of sensibility. In regard to the last objection, also a surmise, it may be observed, that the sensibility of the larynx is probably derived from its investing membrane, supplied by other nerves, the eighth pair affording branches to its movable cartilages and muscles. If this view be correct, it tends to assimilate the eighth with the portio dura of the seventh nerve, as to their properties and functions.

Upon a review of all circumstances lately disclosed by experiments upon the nerves, it seems to be clearly and satisfactorily made out, that, wherever we can trace nerves sufficiently near their origins to find them alone and unconnected with others, they indicate distinct and separate properties; that is to say, a nerve which influences muscular action does not receive and convey impressions of feeling, and one that partakes of the latter property does not influence muscular motion: and this distinction seems to be governed by the peculiar functions of those parts or organs separately and individually belonging to them, to which different nerves are appropriated; and thus the full exercise of nervous influence causes all the functions of an organ to play, whilst a suspension or destruction of power in any one nerve, by forcible pressure or division of its original trunk, robs that organ which it may supply with branches of one of its functions, without injury to another. Hence, where more than one function is performed by any part of the body, it receives the branches of so many nerves as correspond with the number of its functions, and which, although they may travel together in the same sheath, possess different properties independently of each other, according to the functions of the different textures over which they are finally distributed.

Cases of hemiplegia, and other sources of paralytic affection from accident or disease, frequently illustrate the distinction, and coincide with the phenomena of the above and similar experiments.

*Great Marlborough street; April* 1823.

